# Study of Atmospheric Bacteria

**Published:** 1887-03

**Authors:** 


STUDY OF ATMOSPHERIC BACTERIA.—N. Kelguish has made a study of the bacteria of the air, his investigations extending over a period from September, 1885, to August, 1886, inclusive. During this time 186 examinations of the air were made, and 846 cultivations obtained, from which it was determined that the air of St. Petersburgh contained on an average 4,500 microbes per cubic meter. In winter the external air is purest, containing 2,000 microbes per cubic metre, while that of hospitals contained most germs, 5,350 per cubic metre. The observations of Kelguish confirm those of Miguel, who claims that the external air is purest in winter, while the opposite is true of the air of closed spaces. He also found that the air of medical wards of hospitals is purer than that of surgical wards, being in the ratio of 2,606 germs per cubic metre in the former to 3,488 in the latter. He also showed the great superiority of heated chimneys as a means of ventilation, over the ordinary air-shafts, as the former destroy germs while the latter only remove them.—*Revist. de Cientias Med.*

